# Thymol Dentifrice

**Published:** 1891-10

**Authors:** 


### Thymol Dentifrice.

R. Precipitated chalk........................xv ounces.
Soap, powdered..............................j ounce.
Saccharin.......................................x grains.
Thymol..........................................xv grains.
Camphor.........................................xxx grains.
Vanillin..........................................v grains.
Oil of rose......................................vj drops.

Rub the camphor and thymol together in a mortar, and warm gently so as to render the mixture liquid; then add the chalk in small portions at a time, reserving about one ounce; next add the other ingredients, the perfumes being first separately rubbed with the remainder of the chalk.—*Chemist and Druggits.*

